# The Impact of the Expression Level of Intratumoral Dihydropyrimidine Dehydrogenase on Chemotherapy Sensitivity and Survival of Patients in Gastric Cancer: A Meta-Analysis

**DOI:** 10.1155/2017/9202676

**Published:** 2017-01-31

**Authors:** Cong Zhang, Hongpeng Liu, Bin Ma, Yongxi Song, Peng Gao, Yingying Xu, Dehao Yu, Zhenning Wang

**Affiliations:** ^1^Department of Surgical Oncology and General Surgery, The First Hospital of China Medical University, Shenyang 110001, China; ^2^Department of Breast Surgery, The First Hospital of China Medical University, Shenyang 110001, China

## Abstract

The potential impact that the intratumoral expression level of dihydropyrimidine dehydrogenase (DPD) has on chemotherapy sensitivity and long-term survival for gastric cancer (GC) patients remains controversial; therefore, this study seeks to clarify this issue. Our meta-analysis was performed using Review Manager (RevMan) 5.3 software. In vitro drug sensitivity tests, correlation coefficients between sensitivity to 5-fluorouracil (5-FU), and expression levels of intratumoral DPD were used as effective indexes to analyse. Overall survival (OS) and progression-free survival (PFS) were used as endpoints for patient outcome, and hazard ratios (HRs) and 95% confidence intervals (CIs) were noted as measures of effect. There were 15 eligible studies including 1805 patients for the final analysis. The analysis revealed a statistically significant difference between the expression level of intratumoral DPD activity, DPD mRNA levels, and sensitivity to 5-FU in GC patients, with high expression levels of intratumoral DPD resulting in low sensitivity to 5-FU. However, no matter what therapeutic regimens were used, there was no significant difference for patient outcomes between high and low DPD expression groups, either in OS or in PFS. In conclusion, high levels of intratumoral DPD expression have a negative impact on sensitivity to 5-FU in GC patients, but no prognostic value for long-term survival was uncovered.

## 1. Introduction

The fourth most common malignant tumor type, gastric cancer (GC), is the second leading cause of cancer-related deaths worldwide [1, 2]. Due to a paucity of early symptoms, GC often reaches advanced stages by the time of discovery [3]. D2 radical resection is the standard treatment for advanced gastric cancer (AGC) patients; however, there is still no standard effective chemotherapy regimen for AGC [4–6]. Fluorouracil-based chemotherapy regimens have been widely used as first-line treatments for GC patients [7, 8]. 5-FU and S-1 are the most widely used fluorouracil and there are some differences between their components and pharmacological effects. S-1 is a new oral antitumor drug and consists of tegafur (FT) and the following two types of biological modulators: 5-chloro-2,4-dihydroxypyrimidine (CDHP) and potassium oxonate (Oxo) with molar ratio of 1 : 0.4 : 1, and it was first used in clinical practice in 1999 [9]. FT is a prodrug of 5-FU, and under the effect of cytochrome P-450 in the liver, it can be metabolised into 5-FU [6]. However, due to the significant heterogeneity in GC, there are wide discrepancies in the effect of the same fluorouracil-based regimens between patients [10, 11]. Consequently, it is important to seek a biomarker to evaluate which patients will most benefit from fluorouracil-based regimens and to estimate the long-term outcome of GC patients.

Dihydropyrimidine dehydrogenase (DPD) is the rate-limiting enzyme in the catabolism of fluorouracil and its derivatives [12, 13]. DPD is widely expressed in various tissues around the human body, with high activity in liver tissue and peripheral blood mononuclear cells (PBMC), as well as in tumor tissues [14]. It is reported that fluorouracil decomposition is accelerated in patients with increased DPD activity, resulting in resistance to fluorouracil-based therapy, and in patients with DPD deficiency, serious chemotherapeutic toxicity is observed [15–17]. However, for different fluorouracil, such as 5-FU and S-1, the effect of DPD on them will be discrepant because of the existence of CDHP. As a component of S-1, CDHP is a strong DPD inhibitor; therefore, S-1 will generate higher concentration of 5-FU in both the blood and tumor tissue [18]. These effects have made DPD as a biomarker for GC patient an attractive field of study. Some studies reported that DPD had the predictive value on sensitivity to 5-FU and prognostic value on long-term survival [14, 19]. However, other studies found no similar role of DPD or even reached the opposite conclusion [1, 20]. Therefore, the predictive and prognostic value of DPD for GC remains enigmatic.

With the development of biomarker research, finding highly sensitive and specific biomarkers is becoming more important and popular [21–23]. In this meta-analysis, we focus on the relationship between expression levels of intratumoral DPD and sensitivity to 5-FU and outcome of GC patients, with the intent to establish the value of DPD as a biomarker.

## 2. Materials and Methods

### 2.1. Literature Search

The electronic databases of PubMed and Embase were systematically searched for literature published through August 2016 to find all eligible studies for use in this meta-analysis. We conducted this retrieval using the search terms “dihydropyrimidine dehydrogenase”, “DPD”, “DPYD”, “gastric”, and “stomach”. The language of the articles was restricted to English. The reference lists of the identified studies were meticulously searched to identify additional relevant studies.

### 2.2. Inclusion Criteria

To make our studies reliable and accurate, we used the following inclusion criteria: (1) patients were histologically diagnosed with gastric adenocarcinoma; (2) specimens used to detect the expression level of DPD were obtained by endoscopic biopsy or postoperative samples prior to chemotherapy; (3) the correlation coefficients between the expression level of intratumoral DPD and sensitivity to 5-FU could be found or calculated in the relevant studies; (4) the information on survival was sufficient to calculate HR.

### 2.3. Exclusion Criteria

Studies containing the following criteria would be excluded: (1) patients received neoadjuvant chemotherapy or radiotherapy; (2) the sample size of a single study was less than 20; (3) if there was more than one article from the same author with samples from the same patient population, only the most recent and comprehensive study was eligible for this analysis and the others were excluded.

### 2.4. Data Extraction

The following data were independently extracted from each study by the two authors (Cong Zhang and Hongpeng Liu): the name of first author, publication year, country, sample size, study type, disease stage, chemotherapy regimen, the measuring method of DPD, cut-off value of DPD, follow-up times, survival data (HR was provided or survival curve was available for us to extract HR), and the correlation coefficients between expression levels of intratumoral DPD and sensitivity to 5-FU. Any inconsistencies in the data extracted by the two authors were resolved through consultation and discussion.

### 2.5. Statistical Methods

This meta-analysis was performed by Review Manager (RevMan) 5.3 software (The Nordic Cochrane Centre, Copenhagen, Denmark). The correlation coefficients from studies that researched chemotherapy sensitivity were transformed to Fisher's *z* values. According to the generic inverse variance method, we obtained the pooled Fisher's *z* values and 95% CIs and transformed them back to correlation coefficients using published corresponding formulas [24–27]. We used HRs and 95% CIs as effect values to research the relationship between the expression level of intratumoral DPD and survival of GC patients. The survival information was presented using OS and PFS. If they were available in the articles, HRs and their 95% CIs were directly pooled. If the HRs of univariate and multivariate analysis were both given, we only used the latter. When they were not given explicitly, the method designed by Tierney et al. was adopted [28]. Forest plots were used to show the pooled results of Fisher's *z* values and HRs.

Heterogeneity tests were performed by the index of *I*^2^ and *Q* tests. *I*^2^ values range from 0 to 100. *I*^2^ > 50% and/or a *P* value of *Q* test <0.10 indicated that large heterogeneity existed, so the random effects model was applied; otherwise we used the fixed effects model [29, 30]. Because large heterogeneity for survival analysis was seen throughout this meta-analysis, only the random effects model was used to pool the HRs. A two-sided *P* value < 0.05 was considered to be statistically significant. Finally, a funnel plot was utilized to test for publication bias.

## 3. Results

### 3.1. Characteristics of Eligible Studies

**Figure 1:**
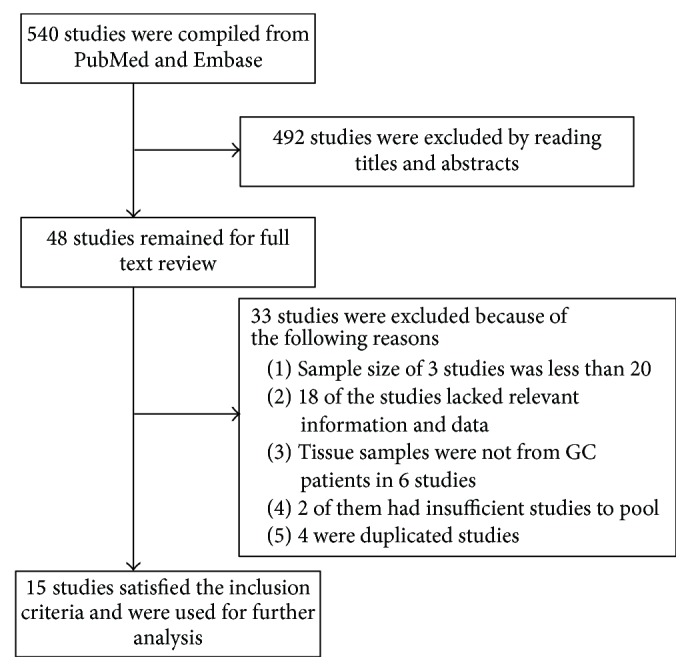
Flow chart for selecting studies.

Our initial search compiled 220 articles from PubMed and 320 articles from Embase. After reading the titles and abstracts, 48 articles were found to meet the inclusion criteria. When we obtained the full text of these articles for the further analysis, 33 studies were excluded and only 15 were selected for final inclusion. Reasons for exclusion from the analysis are listed in Figure 1. Of these 15 studies [13, 15, 19, 20, 31–41], 14 were from Japan and the one remaining was from Korea. All of them were retrospective studies. Because of the late stage, fluorouracil-base regimens such as S-1 monotherapy or combination chemotherapy were utilized in some studies [19, 31–33, 35, 36], and 5-FU was used to detect chemotherapy sensitivity in all 5 studies. Radioenzymatic assays, reverse transcriptase polymerase chain reaction (RT-PCR), and immunohistochemistry (IHC) were applied to measure DPD activity, mRNA expression, and protein levels, respectively. Tetrazolium-based colorimetric assays (MTT), in vitro ATP assays, and histoculture drug response assays (HDRA) were used to estimate sensitivity to 5-FU. In one study, the indicator to assess sensitivity to 5-FU was different from others, yet we transformed the results into compatible data [39]. For IHC results, we considered patients with DPD negative expression as low DPD expression group and those with positive expression as high DPD expression group. Ultimately, the total sample size from all eligible studies was 1805 (median: 75, range from 32 to 401). Other characteristics such as the methods for choosing the cut-off value of DPD, chemotherapy regimens, and follow-up times are listed in the Table 1.

### 3.2. The Predictive Value of DPD for Chemotherapy Sensitivity

**Figure 2:**
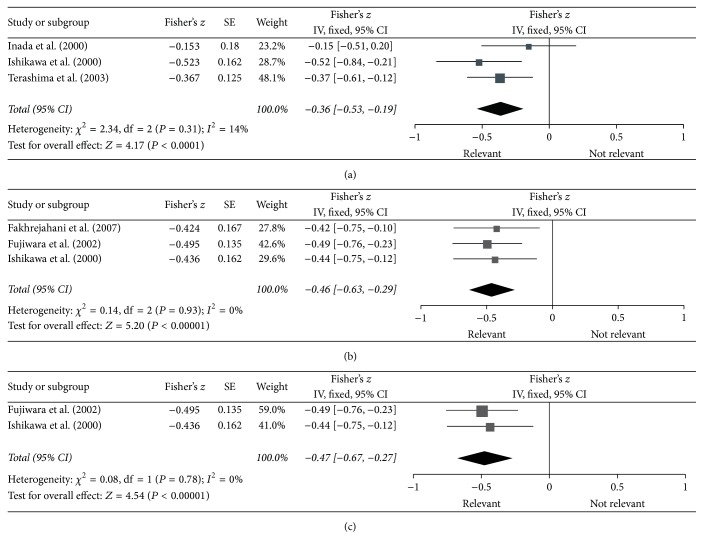
Forest plot of pooled Fisher's *z* value for sensitivity to 5-FU: (a) DPD activity; (b) DPD mRNA; (c) DPD mRNA when excluding a study.

For three studies measuring DPD enzymatic activity, pooled Fisher's *z* value was −0.36 (95% CI: −0.53 to −0.19, *P* < 0.0001) (Figure 2(a)). In studies that measured DPD mRNA levels, pooled Fisher's *z* value was −0.46 (95% CI: −0.63 to −0.29, *P* < 0.00001) (DPD activity and mRNA were both measured in one study) (Figure 2(b)). Statistically significant differences were observed. Because the heterogeneity in both subgroups was small (*I*^2^ = 14% and *I*^2^ = 0), we adopted a fixed effects model to pool the results. The final correlation coefficients transformed from pooled Fisher's *z* values were −0.35 (95% CI: −0.49 to −0.19) and −0.43 (95% CI: −0.56 to −0.28), respectively, indicating that a statistically significant relationship existed, with high expression levels of intratumoral DPD resulting in low sensitivity to 5-FU. In one study, samples were divided into differentiated and undifferentiated-type tumors, but the correlation between expression levels of intratumoral DPD mRNA and sensitivity to 5-FU in all samples was not experimentally examined, and the remaining two studies that measured DPD mRNA level were also pooled when excluded it. Pooled Fisher's *z* value was −0.47 (95% CI: −0.67 to −0.27, *P* < 0.00001) (Figure 2(c)) and the final correlation coefficient was −0.44 (95% CI: −0.59 to −0.26), indicating that this relationship was also statistically significant.

### 3.3. The Prognostic Value of DPD for Outcome of Patients

**Figure 3:**
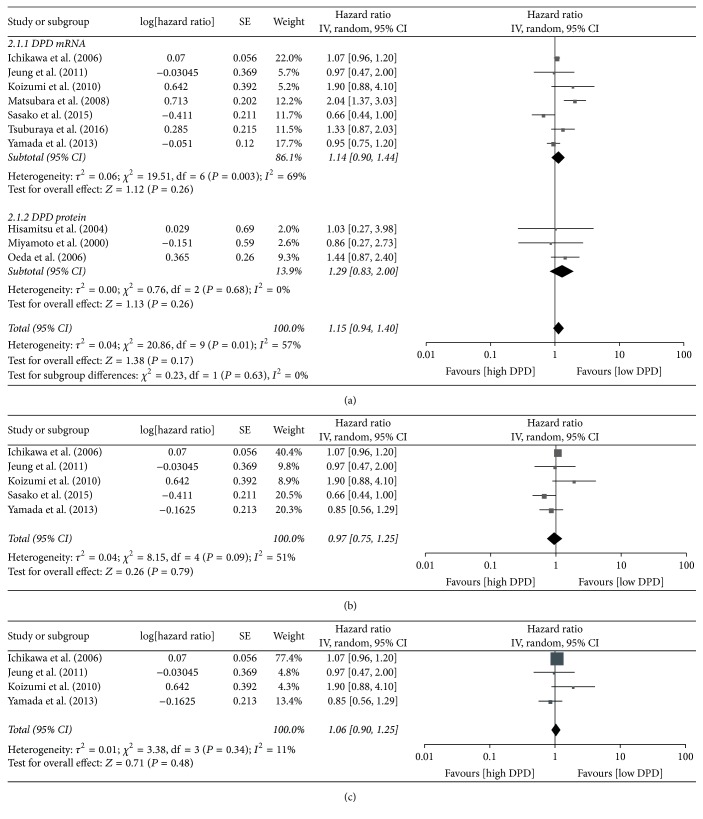
Forest plot based on outcome of patients: (a) OS; (b) OS for 5 studies treated by S-1 monotherapy; (c) OS for 4 studies treated by S-1 monotherapy that were late stage.

**Figure 4:**
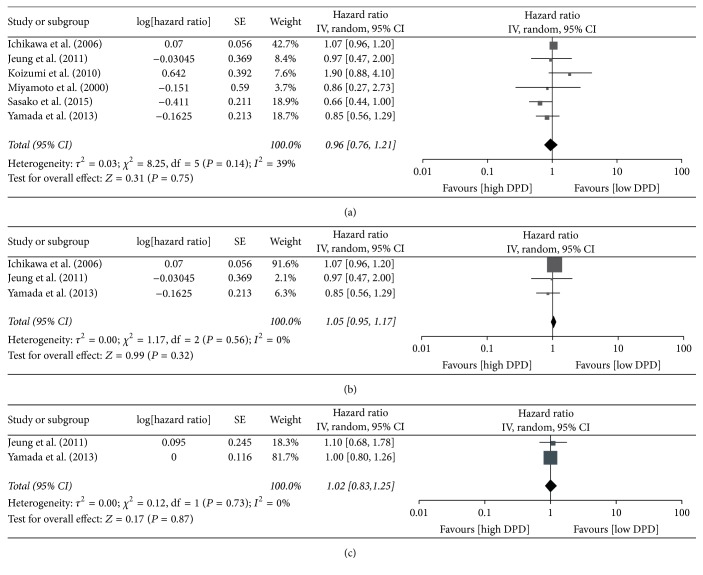
Forest plot for (a) 6 studies using S-1 as chemotherapy regimen; (b) 3 studies using median cut-off value; (c) 2 studies using PFS as outcome indicator.

**Figure 5:**
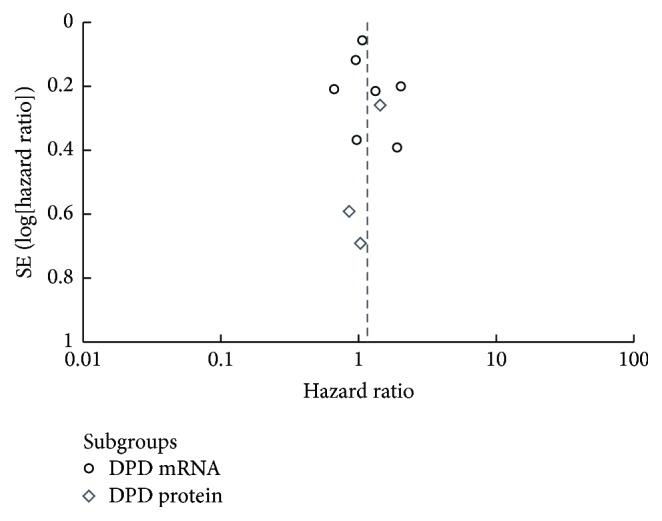
Funnel plot for 10 studies using OS as outcome indicator.

We pooled HR for OS throughout 10 studies that researched patient outcome, amassing a pooled result of 1.15 (95% CI: 0.94 to 1.40, *P* = 0.17). High heterogeneity was observed (*I*^2^ = 57%), so the random effects model was used (Figure 3(a)). Although the results indicated that high expression levels of DPD in tumor tissue trended toward a negative impact on GC patient outcome, it was not statistically significance. Subgroups were created according to the method used for measuring DPD levels, and the pooled HRs for OS of different levels of DPD mRNA and DPD protein were 1.14 (95% CI: 0.90 to 1.44, *P* = 0.26) and 1.29 (95% CI: 0.83 to 2.00, *P* = 0.26), respectively. The results still had no statistical significance. For the seven studies that measured DPD mRNA levels, five of them received S-1 monotherapy as their chemotherapy regimen, so we also pooled HRs for OS across these five studies, and the resulting HR was 0.97 (95% CI: 0.75 to 1.25, *P* = 0.79) (Figure 3(b)). Four of the five studies included patients with late disease stage, including metastatic, recurrent, or unresectable GC, where surgery could not be performed and S-1 monotherapy was adopted. The pooled HR for OS of these four studies was 1.06 (95% CI: 0.90 to 1.25, *P* = 0.48) (Figure 3(c)). As above, in the latter two analyses, we found no statistically significant correlation between the expression level of intratumoral DPD and OS of GC patients. In these 10 studies, six of them are using S-1 as chemotherapy regimen, and we also pooled the HR for OS in order to distinguish S-1 from other 5-FU based treatments. The result of the pooled HR was 0.96 (95% CI: 0.76 to 1.21, *P* = 0.75) (Figure 4(a)). We did not find the prognostic role of DPD on patient outcome after S-1 treatment. In specified analyses, Figures 3(b) and 3(c), we eliminated the studies that are far from the median cut-off value for consistency, and these two pooled results were the same. The pooled HR for OS was 1.05 (95% CI: 0.95 to 1.17, *P* = 0.32) (Figure 4(b)). The results also had no statistical significance. The funnel plot of 10 studies using OS as the outcome indicator was showed in Figure 5. No significant asymmetry was observed, so we concluded that there was no obvious publication bias. HR for PFS was available to pool in two of the 10 studies, and the result of the pooled HR was 1.02 (95% CI: 0.83 to 1.25, *P* = 0.87), with no significant difference and no clear correlation between the expression level of intratumoral DPD and PFS for GC patients (Figure 4(c)).

## 4. Discussion

With the development of individualized precision medicine, more researchers have begun to look for biomarkers to guide therapy or estimate the outcome of patients with GC. Fluorouracil-based chemotherapy regimens, especially S-1 monotherapy or combination chemotherapy, have been applied clinically to treat GC for many years. As the most commonly used fluorouracil agent, S-1 can be metabolised into 5-FU in human body and has higher response rate than 5-FU or capecitabine with mild side effects. The response rate for S-1 in AGC is 26%–49%, so it has been widely applied clinically [42, 43]. With the extensive application of fluorouracil-based chemotherapy regimens, especially S-1, in the treatment of GC, it is important to find a biomarker to select for patients who may most benefit from its use. This is especially critical due to the high heterogeneity present between patients with the same stage of GC, resulting in vastly different outcomes to fluorouracil-based treatment regimens. As the rate-limiting enzyme in the catabolism of fluorouracil, DPD plays an important role in GC, and its intratumoral expression has been demonstrated to associate with the degradation of fluorouracil and its related toxic effects [12, 33]. However, whether DPD has impact on chemotherapy sensitivity, response rate to drugs, or long-term survival remains controversial. Several studies have researched the role of DPD in GC with contrasting conclusions. Therefore, we performed this meta-analysis in order to clarify whether the expression level of intratumoral DPD correlates with chemotherapy sensitivity and outcome in GC patients, especially for those receiving fluorouracil-based regimens.

Through this meta-analysis, we found a statistically significant negative correlation between DPD and sensitivity to 5-FU, with high expression levels of intratumoral DPD resulting in low sensitivity to 5-FU. However, we found no significant impact of DPD expression levels on the outcome of GC patients whether they received fluorouracil-based regimens or not, and these same results were consistent in analyses of subgroups segregated by different methods used for measuring DPD. Therefore, we provide evidence that the expression level of intratumoral DPD may impact patient sensitivity to 5-FU, but it does not has the prognostic value on patient outcome.

In our meta-analysis, in vitro sensitivity to 5-FU was shown to significantly associate with the expression level of intratumoral DPD, with tumors with high DPD expression leading to 5-FU resistance. DPD is the initial and rate-limiting enzyme for fluorouracil catabolism, and 5-FU is mainly decomposed by it in the liver and other tissues [6]. Therefore, the expression level of DPD throughout the human body affects the degradation of 5-FU. DPD expression is observed in tumor tissue, and Toriumi et al. found that tumors with high expression of DPD mRNA were resistant to 5-FU treatment [7]. Terashima et al. also demonstrated a weak inverse correlation between DPD activity and sensitivity to 5-FU, with tumors with high DPD activity resulting in 5-FU resistance [44]. Furthermore, Terashima et al. found the same inverse relationship to apply to DPD protein expression [45]. Park et al. discovered that DPD might be an important factor to regulate sensitivity to 5-FU even in GC cell lines [46]. However, in some studies, DPD mRNA expression levels did not correlate with 5-FU chemosensitivity, suggesting that the reasons might be attributed to the different methods used for measuring DPD and chemosensitivity testing [47].

Our study found no prognostic value of DPD expression levels for long-term survival of GC patients. The impact of DPD on outcome has been studied by many researchers, but the results are inconsistent, even opposing. Matsubara et al. posited a relationship between gene expression levels of DPD and OS in AGC patients, with high DPD indicating poor survival [19]. This was confirmed by Terashima et al. showing that patients with high DPD activity have poorer OS when treated with 5-FU-based therapy [44]. However, Tahara et al. found that levels of DPD protein had no impact on the median survival time (MST) of patients [48]. This finding was corroborated by results from Shimizu et al. and Moiseyenko et al., with both suggesting that DPD had no prognostic value on outcome for GC patients [49, 50]. In stark contrast to other studies, Kim et al. found that low expression levels of intratumoral DPD associated with poor disease-free survival (DFS), and they suggested that low DPD expression levels might be related to increased S-1 toxicity and decreased tolerance to S-1, resulting in patients with low intratumoral DPD expression having poor DFS [1]. In addition, the study created by Sasako et al. also got the similar conclusion with Kim et al. that high DPD gene expression was connected with better efficacy of postoperative adjuvant chemotherapy with S-1 for GC [34]. The samples they used to analyse were from the Adjuvant Chemotherapy Trial of S-1 for Gastric Cancer (ACTS-GC) and this study had the highest number of patients with better design methods, so it was a high quality research and considered to be more reliable. They suggested that it was because S-1 had some effects not owned by other fluoropyrimidines such as gimeracil, a potent DPD inhibitor could avoid the resistance to 5-FU when DPD activity was high. Beyond that, they considered that DPD might have the roles in the inhibition of recurrence by S-1 with thymidylate synthase (TS) together. Finally, they suggested that we could select chemotherapy regimens according to the expression level of DPD in the tumor tissue. Even though it was a high quality research, the final consequence they obtained was from a single cohort and the result was different from many other researches, and our meta-analysis did not reveal the prognostic roles of DPD for patient outcome. Therefore, DPD has remained unusable as a biomarker to estimate the outcome of GC patients.

An interesting result illustrated in this meta-analysis is that DPD has a significant predictive value for sensitivity to 5-FU, but not for patient survival. The ability to increase survival rate is of great importance for all cancer patients. The cause of this phenomenon might be found in distinguishing between 5-FU and S-1. Throughout the studies included in this analysis, 5-FU was the most common drug used for researching chemotherapy sensitivity, but S-1 was widely used to perform survival analyses. Koizumi et al. demonstrated a lack of prognostic value for DPD on patient outcome when treated with S-1, and they suggested that the antitumor effect of S-1 was also favourable even in tumors with high DPD expression [33]. A similar result was found by Ichikawa et al., who posited that the result may be due to CDHP, a part of S-1, which could inhibit the activity of DPD so that the concentration of fluorouracil could be maintained at high levels for long periods of time [31]. However, whether DPD has an impact on GC patients without 5-FU or S-1 based therapy remains unresolved.

Fakhrejahani et al. found that the expression level of DPD gene was higher in undifferentiated than differentiated tumors for GC [38]. The same result was found by Ichikawa et al., and they also demonstrated that DPD gene expression level was lower in differentiated type tumor tissue than normal stromal tissue, but there was no difference in undifferentiated type [51]. Hisamitsu et al. made a point that DPD expression level in cancer cells but not in stromal cells could predict the efficacy of fluorouracil chemotherapy for GC patients [13]. Due to the different differentiation degree between diffuse and intestinal type tumors, differences may exist in DPD expression level; therefore, the roles of DPD will be different between these two type tumors. However, most of the studies lacked relevant data such as sensitivity to drugs and survival information and we could not analyse the different roles of DPD between diffuse and intestinal type tumors; we will focus on this problem in the future.

The enzymes involved in the metabolism of fluorouracil include not only DPD, but also others such as orotate phosphoribosyltransferase (OPRT), thymidylate synthase (TS), and thymidine phosphorylase (TP). The impact of these enzymes on GC patients has been noticed by other researchers. Hu et al. found that high expression levels of TS might indicate poor outcome for GC patients treated with fluoropyrimidine-containing chemotherapy regimens [52]. In Sakurai et al., the role of OPRT in GC patients was researched and the prognosis of patients was found to be better with high expression levels of OPRT [53]. Nishina et al. demonstrated that the ratio of TP to DPD expression levels associated with a positive impact on response rate and survival for GC patients [54]. It should be noted that some of these studies had small sample sizes and that there was large heterogeneity present within the results. This meta-analysis found no significant prognostic value for GC patient outcomes using DPD alone, but DPD might play a predictive and prognostic role when grouped with others. Future studies should probe whether combinations of these enzymes could have a significant predictive and prognostic effect, including a systematic study combining all four enzymes involved in the catabolism of fluorouracil to define possible predictive and prognostic values associating their expression with chemotherapy sensitivity and long-term survival in GC patients.

This analysis had the following limitations. First, all of the studies incorporated into this meta-analysis were retrospective studies, and most of them were from Japan, with the remaining one which was coming from Korea. Because of the limitations of region and ethnicity, our study results might not apply to all populations. Second, there was no standard method for measuring DPD; we could monitor DPD mRNA, protein, or activity levels, but it is not clear which is most effective. Third, the cut-off value for expression of DPD was not defined, so individual studies adopted different methods to define it. Fourth, sample sizes of the studies included in this meta-analysis were often inadequate, with sample sizes in more than half of the studies including less than 100 patients. For five studies researching chemotherapy sensitivity, correlation coefficients in three of them were Spearman's rank correlation coefficient, but in the other two studies, we only know that linear regression analysis was performed. Finally, methodological assessment of the included studies was not performed by us, since there was no standard method for detecting the expression level of DPD. In general, these limitations in our study were innate and could not be eliminated through analytic techniques.

## 5. Conclusions

In conclusion, there is currently not enough evidence for DPD to become a biomarker in GC patients, especially for patient outcome. However, we find that the expression level of intratumoral DPD has a significant impact on sensitivity to 5-FU, but it is not yet advised to base decisions regarding fluorouracil-based treatment regimens on the expression level of DPD. Further research is needed before DPD can act as a guide for clinical medication therapies or as a biomarker to estimate long-term survival of GC patients.

## Figures and Tables

**Table 1:** Basic characteristics of eligible studies.

First author	Year	Country	Sample size	Disease stage	Drugs	Method	Cut-off value	HR for OS (95% CI)	HR for PFS (95% CI)	Correlation coefficient	Drug sensitivity test	Follow-up times
Ichikawa [31]	2006	Japan	59	Late	S-1	RT-PCR	Median	1.08 (0.97 to 1.21)				2 years
Jeung [32]	2011	Korea	75	Late	S-1	RT-PCR	Median	0.97 (0.47 to 2.00)	1.1 (0.68 to 1.78)			4 years
Koizumi [33]	2010	Japan	65	Late	S-1	RT-PCR	*χ* ^2^: 1.68 × 10^−3^	1.9 (0.88 to 4.09)				3 years
Matsubara [19]	2008	Japan	137	Locally advanced or late	S-1, 5-FU, cisplatin + S-1 or irinotecan	RT-PCR	*χ* ^2^: 1.18 × 10^−3^	MA: 2.04 (1.37 to 3.02)				7 years
Sasako [34]	2015	Japan	401	Stage II, III, R0 resection	S-1	RT-PCR	33.3rd, 50th, or 66.7th percentiles	MA: 0.66 (0.44 to 1.01) (33.3rd)				5 years
Yamada [35]	2013	Japan	304	Late	5FU, cisplatin + irinotecan, S-1	RT-PCR	Median	All: 0.95 (0.75 to 1.20); S-1: 0.85 (0.56 to 1.29)	1.00 (0.80 to 1.26) (all); 0.93 (0.63 to 1.38) (S-1)			3 years
Tsuburaya [40]	2016	Japan	106	Locally advanced or late	S-1 or S-1 + irinotecan	RT-PCR	Median	MA: 1.33 (0.87 to 2.02)				3 years
Hisamitsu [13]	2004	Japan	111	T3 stage, R0 resection	UFT	IHC	Percentage^b^: 5%	1.03 (0.27 to 3.98)				5 years
Miyamoto [36]	2000	Japan	41	Late	S-1	IHC	Intensity^c^ Negative 0,1 Positive 2,3	0.86 (0.27 to 2.73)				2 years
Oeda [37]	2006	Japan	221	Stages I to IV	UFT, MMC + 5-FU	IHC	Proportion^d^: 10%	1.44 (0.86 to 2.40)				5 years
Fakhrejahani [38]	2007	Japan	87	Stages I to IV	5-FU	RT-PCR				−0.401	HDRA	
Inada [20]	2000	Japan	34^a^	Locally advanced	5-FU	Enzymatic assay				−0.152	HDRA	
Ishikawa [39]	2000	Japan	41	Locally advanced	5-FU	RT-PCR and enzymatic assay				−0.41 (mRNA) −0.48 (activity)	MTT assay	
Terashima [15]	2003	Japan	67	Stages I to IV	5-FU	Enzymatic assay				−0.351	ATP assay	
Fujiwara [41]	2002	Japan	58	Stages I to IV	5-FU	RT-PCR				−0.458	ATP assay	

*Note*. 5-FU: 5-fluorouracil; UFT: uracil/tegafur [4 : 1]; MMC: mitomycin C; IHC: immunohistochemistry; RT-PCR: reverse transcriptase polymerase chain reaction; HR: hazard ratio; OS: overall survival; CI: confidence interval; PFS: progression free survival; MA: multivariate analysis; HDRA: histoculture drug response assay; MTT: tetrazolium-based colorimetric assay; *χ*^2^: maximal *χ*^2^ method.

^a^There was a patient with triple advanced gastric cancer. ^b^Percentage of stained cells to the total tumor cells. ^c^The intensity of immunohistochemistry staining. ^d^Proportion of cancer cells that were positively stained.
